# Lot quality assurance sampling to assess coverage and compliance following mass drug administration to eliminate lymphatic filariasis in Fiji: A methodological approach

**DOI:** 10.1371/journal.pone.0238622

**Published:** 2020-09-18

**Authors:** Milika Rinamalo, Lorenzo Pezzoli, Mike Kama, Eric Rafai, Ilisapeci Kubuabola, Mosese Salusalu, Sung Hye Kim

**Affiliations:** 1 Ministry of Health and Medical Services, Dinem House, Suva, Republic of Fiji; 2 Epidemiology Consultant, London, United Kingdom; 3 College of Medicine, Nursing and Health Sciences, Fiji National University, Suva, Republic of Fiji; 4 Department of Tropical Disease Biology, Liverpool School of Tropical Medicine, Liverpool, United Kingdom; Helen Keller International, SIERRA LEONE

## Abstract

**Background:**

Assessing the quality of mass drug administration (MDA) rounds is a key component of lymphatic filariasis (LF) elimination programs. Routine collection of administrative coverage is unreliable, especially when pockets with low program coverage exist. To address this gap, we used lot quality assurance sampling (LQAS) following the 10th annual LF-MDA round in Fiji to explore whether there was any area in which target coverage was not reached. We also assessed the level of drug compliance and satisfaction with the LF-MDA implementation strategy.

**Methodology/principal findings:**

We conducted a cross-sectional household survey in 3 divisions of Fiji. For LQAS, we defined 19 lots in 7 medical areas of the Suva sub-division and another 12 sub-divisions in the Central, Northern, and Eastern Divisions. A sample of 16 randomly selected household members was taken un each lot. We defined our decision rule as follows: if more than 1 person in a given lot did not swallow the medication, coverage was considered inadequate, i.e. less than 80%.

Of the 7 lots in Suva sub-division and 12 lots in the 3 divisions, five and two lots, respectively, were identified as having inadequate coverage. The overall program coverage estimated from 304 samples was 92%, which was higher than the reported administrative coverage of 82%. About 98% of interviewees were offered the medication and 96% swallowed it. Non-participation arose from insufficient information on how to obtain the drugs. At least 92% were satisfied with the LF-MDA implementation strategy.

**Conclusions:**

Areas of low program coverage with results discordant with the reported administrative coverage existed in both urban and rural settings. Drug compliance and satisfaction were high, even after repeated rounds. We recommend increasing efforts to deliver the service in those areas with inadequate program coverage, as well as conducting timely coverage assessment through LQAS for corrective action.

## Introduction

Lymphatic filariasis (LF) is a mosquito-transmitted parasitic disease caused by the filarial nematodes *Wuchereria bancrofti*, *Brugia malayi* and *Brugia timori*. It affects an estimated 120 million people throughout the tropics and there are as many as 36 million cases of hydrocoele and lymphoedema [1, 2]. In 1997, the 50th World Health Assembly (WHA) approved a resolution calling for the elimination of LF as a public health problem by 2020 (WHA50.29). Shortly after, the Global Program to Eliminate Lymphatic Filariasis (GPELF) was established. GPELF aims to achieve the goal through annual rounds of single-dose mass drug administration (MDA) of albendazole (one fixed dose of 400 mg) with diethylcarbamazine citrate (DEC, 6 mg/kg body weight) for at least 5–6 years [3], for those older than 2 years in countries where onchocerciasis is not co-endemic. The current recommendation is that each LF-MDA round should achieve epidemiological coverage of at least 65% of the at-risk population [4, 5].

The Pacific Program for the Elimination of Lymphatic Filariasis (PacELF) is the regional arm of GPELF for the Pacific Island countries and Territories (PICTs) [6]. Sixteen of the 22 countries under PacELF were classified as endemic for LF following baseline prevalence surveys conducted in 2000–2001 [6, 7]. Fiji is endemic for LF and has a total population of 885,000 (2017 estimate) scattered over more than 100 permanently inhabited islands [8]. *W*. *bancrofti*, transmitted by *Aedes polynesiensis*, *Ae*. *pseudoscutellaris* and several other mosquito species, is responsible for LF transmission in that country [3, 9]. *Ae*. *polynesiensis* is of particular concern in the region as there are currently no effective measures for controlling this vector due to a trait called "limitation", that makes it highly efficient in transmitting LF even when the prevalence within the population is low [10].

Historically, Fiji has the highest LF endemicity in the Pacific, and a long history of implementing control measures [3, 9]. The country joined PacELF in 1999 and conducted a nationwide baseline blood survey on a sample of 5,983 people, which showed that 16.6% of the survey participants were positive for circulating filariasis antigen (CFA), tested by the Binax Now® Filariasis Immunochromatographic Test (ICT) of Alere Scarborough (Scarborough, ME) [7]. An LF-MDA round using the combination of DEC (6mg/kg) and albendazole 400mg was commenced in 2002, covering 66% of the population, and was continued until 2006 [7]. An LF prevalence survey in 2007 estimated the national circulating antigen prevalence to be 9.5%, which triggered further consecutive rounds of LF-MDA from 2008 [11]. At the completion of another 5 rounds in 2012, an in-depth review by the national program determined that a major issue was uncertainty about the extent of the actual LF-MDA program coverage reached. Hence, a post-LF-MDA program coverage assessment was proposed to estimate the real coverage in the population including the public’s compliance with the regimen, ideally by sub-division, since the sub-divisions are highly heterogeneous.

Several methods have been proposed for assessing the coverage of public health interventions; of these, the Lot Quality Assurance Sampling (LQAS) technique is useful in making accept/reject decisions as to whether an area has achieved a minimum level of coverage, while requiring a relatively small sample size [12–14]. The LQAS is based on two coverage thresholds, a lower threshold (LT), which is the minimum coverage level considered to be acceptable, and an upper threshold (UT), which is generally the target coverage; it also specifies a lot sample size (N), and a decision value (d) [15]. If the number of untreated individuals detected in the sample N is *≤ d*, the lot is "accepted", meaning that coverage is probably equal to or above the LT. If the number of untreated people is > *d*, the lot is "rejected", meaning that coverage is probably below the UT. Alpha is the probability of “accepting” a lot with low coverage; while beta is the probability of “rejecting” a lot with high coverage [16]. The main advantage of LQAS is the small sample size required to classify lots with regard to coverage levels. Although this classification is binary (accept/reject) and doesn't provide a coverage estimate [17], provided the lots (e.g. sub-divisions) cover a larger area (e.g. a division) and do not overlap, lot data can be aggregated according to a stratified weighted design to estimate coverage in the area [18].

We report on the first national assessment of LF-MDA program coverage in Fiji, which used LQAS as a unique methodologic approach not only to obtain programmatic information on areas with low program coverage but also to explore issues affecting the overall LF-MDA implementation strategy at the national level; this should assist the Ministry of Health and Medical Services (MHMS) in adjusting the strategy towards achieving the elimination goal. The study was conceived and implemented before the global guidelines for coverage assessment and tools for improving the quality of reported MDA data became available [19–21].

## Materials and methods

### Study area

Fiji is an island country located 2,000 km from the northeast of New Zealand, east of Vanuatu, and west of Tonga, in the South Pacific. It is an archipelago of more than 300 islands, of which one third are permanently inhabited by 837,271 residents [8] with a total land area of about 18,300 km2. Up to 87% of the population reside on the two major islands, Viti Levu and Vanua Levu [8].

**Fig 1:**
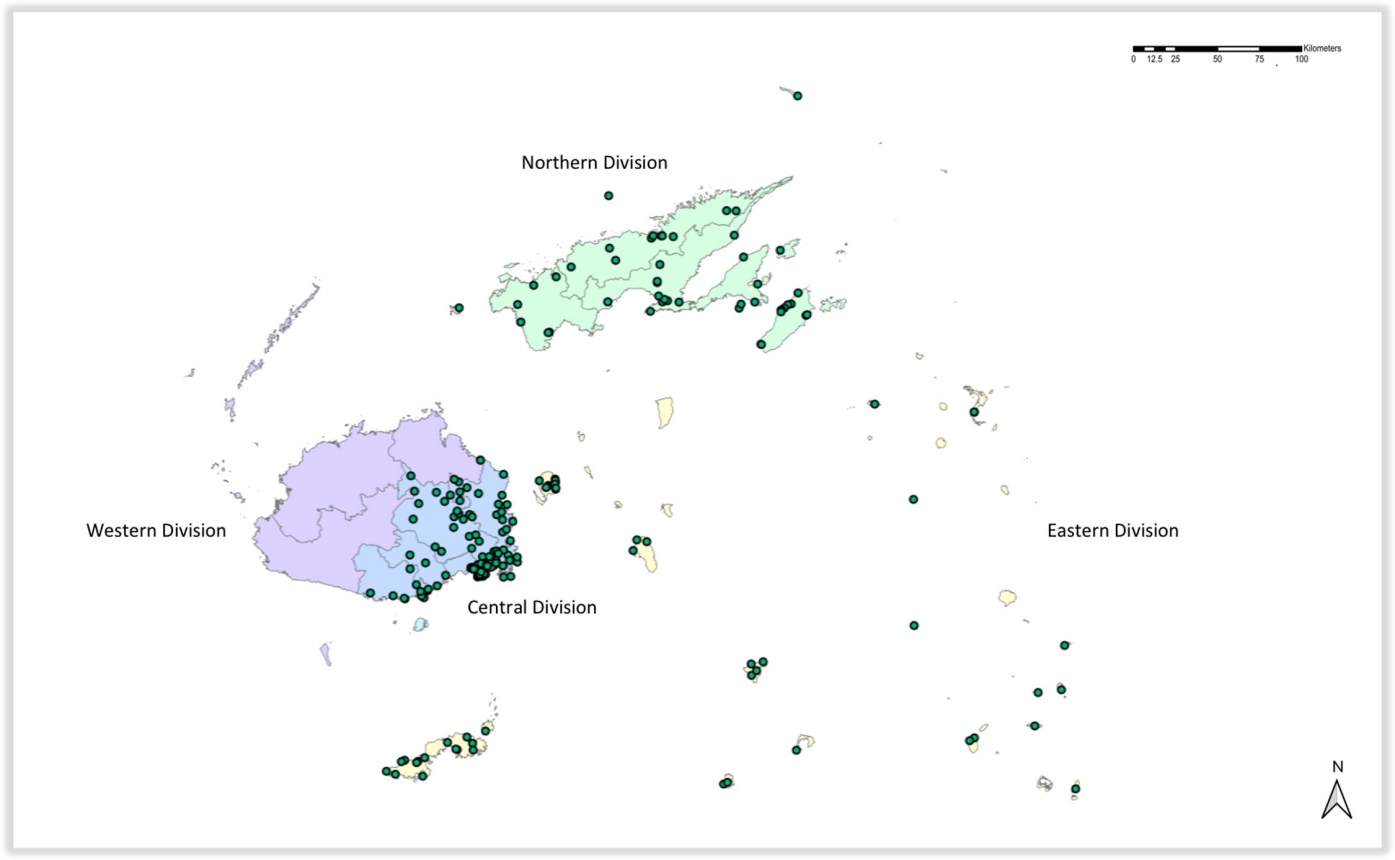
Map of Fiji with 3 Health Divisions with households selected for the coverage assessment of the 10^th^ round of LF MDA. The map was developed using ArcMap 10.4 (ESRI, Redlands, CA) and shapefiles from the GADM database of Global Administrative Areas, ver. 2.8 (gadm.org).

**Fig 2:**
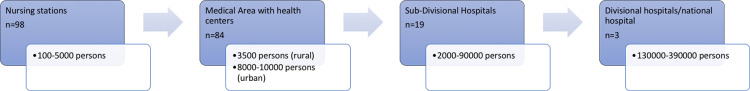
Administrative levels of the health system designated by the Ministry of Health and Medical Services, Fiji [22].

MHMS delivers health services throughout the four Health Divisions, Central, Eastern, Western and Northern [22]. The Western Division consists of the dry western half of Viti Levu, while the Central Division is the wet eastern half. Vanua Levu comprises the Northern Division together with Taveuni Island. The remaining islands are grouped in the Eastern Division (Fig 1). The provision of health services ranges from general to specialized clinical services as well as public health programmes, with health personnel at nursing stations, health centres, and sub-divisional and divisional hospitals (Fig 2) [22]. The Central/Eastern Division is the largest by population size, up to 422,917, and caters to about 100 health facilities, with the majority residing in the Suva sub-division [23]. The Northern Health Divisional office provides health services for 4 sub-divisions and there are about 50 health facilities for the population of 135,282 as of the year 2015 [23]. For this survey, we defined the study area as the same as that targeted by the 10^th^ LF-MDA round, namely the 3 Health Divisions of Fiji (i.e. Central, Northern and Eastern Divisions) (Fig 1).

### Mass drug administration

An annual LF-MDA round was conducted in the three Health Divisions (13 sub-divisions) of Fiji between 26th and 28th October 2012, administering albendazole 400mg and diethylcarbamazine citrate 6mg/kg to individuals over 2 years and not pregnant. The total number of residents in the 3 divisions was 558,199, representing two-thirds of the national population [8]. By this round, all sub-divisions except the Northern Division had received 10 rounds of LF-MDA. For each round, logistic arrangements, training, and supervision were organized by the public health teams based in each sub-divisional Health Offices but coordinated by the national LF elimination program at the Fiji Centre for Communicable Disease Control (FCCDC).

Each sub-divisional health office conducted the training of trainers before the implementation of the LF-MDA round. The trainers were usually public health zonal nurses responsible for any public health initiatives in each nursing zone. The nurses generally recruited and trained the community volunteers who had represented certain localities on LF-MDA procedures, such as registration of household members, offering medicine and completing the forms in the booklets. Each community volunteer registered roughly 125 residents and was tasked with administering medication to up to 100 residents via house-to-house visits during the 3-day “Filariasis Free Weekend”. The registration booklets were distributed to these volunteers by zone nurses together with at least 1 bottle of 100 tablets of 400 mg albendazole and a bottle of 1000 50mg DEC tablets. In addition, zone nurses and other health staff distributed drugs at schools and designated fixed sites, such as health centers, markets, and universities. Directly observed treatment (DOT) was applied to all participants and a fingernail of each person’s left hand was marked with an indelible marker after having swallowed the drugs.

### Study design

The LQAS survey was designed to assess whether there were any areas where LF-MDA program coverage did not reach the predetermined goal at the sub-divisional or medical area level set by the national LF elimination program, and to estimate program coverage and evaluate the overall LF-MDA strategy.

The study area was comprised of 13 sub-divisions, of which there were 5 in the Central and 4 in the Northern Division, along with 4 sub-divisions in the Eastern Division. Each of the sub-divisions in the study area was considered a lot in which a fixed number of individuals would be randomly selected to respond to the survey questionnaire. As for the Suva sub-division, whose population exceeds that of the Northern Division, we considered each of the 7 medical areas as lots. This yielded 19 non-overlapping lots, comprising 12 sub-divisions and 7 medical areas in 3 divisions.

Considering that the reported administrative coverage was 82% for the 3 divisions, the LQAS for the LF-MDA coverage assessment, we set 80% as the lower threshold (the minimum acceptable coverage by the national programme) and 95% as the upper threshold (the desired coverage level). We chose alpha (the risk of "accepting" a lot when the actual program coverage was low) to be lower than beta (the risk of "rejecting" a lot when the actual program coverage was not low), assuming that the former is a greater risk for the population targeted. The resulting sample size (N) was 16, with a decision value (d) of 1, an alpha error of 19% and a beta error of 14% (Sample LQ v1.10; Brixton Health, London, UK) [24] after comparing error levels for sample sizes varying between 14 and 17.

### Survey team organization and data collection procedure

The survey was conducted in March 2013, on average 163 days after the commencement of the 10^th^ LF-MDA round in Fiji. Interviews lasted two to 28 minutes (mean duration: 5.5 minutes).

The survey teams worked in pairs, with at least one female member. The teams attended a 2-day training workshop before the initiation of fieldwork. The training covered the study methodology and included implementation of a small pilot survey. The surveyors spoke both English and Fijian and conducted the survey in a locality familiar to them. Each team was provided with a motor vehicle and tasked to survey a target of one lot of 16 households per day.

Per each lot, 16 sampling points (households) were selected using a multi-stage sampling procedure: first, population size was used to choose the first 16 nursing zones from the list of all nursing zones in each lot that was used by the national programme to estimate the quantity of drugs for the LF-MDA round of the lot. Then, as each nursing zone was further divided into specific localities (street, neighborhood, village, or settlement, etc.), the surveyors selected one locality per nursing zone by simple random sampling. The third stage involved selecting one household at random in that locality. For this purpose, the Family Profile Data register of all families and members who reside in the locality, which is kept by Zone nurses at the Nursing Stations, was used. However, when a Family Profile Data register was unavailable, the surveyors sketched a map of the locality, divided it into sectors of approximately equal sizes, and chose one sector at random until each sector was comprised of around 20 households. Finally, they selected one household at random from the list of 20 households. At the selected household, only one eligible resident (> 2 years and not pregnant) was eventually randomly selected for interview.

Subjects were interviewed by the surveyors using a standardized questionnaire. If a person under the age of 12 was selected, the questionnaire was administered to the immediate guardian in the household. No interview was conducted, if, in the selected household only ineligible subjects were around, such as children under 2 years old or pregnant mothers, for the LF-MDA duration, or no one was contactable on the day of the survey. Instead another household in the same locality was randomly selected.

### Data management and statistical analysis

An Excel 2007 (Microsoft Corporation) worksheet was used to enter data, which was then exported to Stata 12 (StataCorp LP, College Station, TX, USA) for analysis. The overall LF-MDA program coverage was estimated using data from all lots as strata aggregated [25], and the sizes of households and the ratio of the lot population to the total targeted population were used to calculate survey weights in estimating the overall program coverage. Assuming simple random sampling (SRS), 75% coverage, + 5% margin of error and a 95% confidence level, the minimum sample size required to estimate coverage was 289. Hence, we were confident that our sample of 304 (19 multiplied by 16) individuals had higher power and/or precision [26] than the minimum sample needed. In order to achieve 95% confidence intervals (CI) by category at the P<0.05 significance level, F ratios were obtained via adjusted Wald tests to explore whether point prevalence estimates were the same across different categories.

### Ethical considerations

Ethical approval for the survey was granted locally by the National Health Research Review Committee, Fijian MHMS, as part of the standard procedure to evaluate the LF-MDA round. The WHO Regional Office for the Western Pacific Ethics Review Committee (WPRO-ERC) also reviewed and approved the study protocol (2013.21.FIJ.1.MVP). Participation was voluntary, and recruitment was undertaken with an introductory letter about the survey provided to selected households. Written consent was obtained before the survey. Members of the household who were not present for the interview were excluded from the survey.

## Results

### Demographic characteristics of the survey participants

The study included 304 persons, from the same number of households, in three Health Divisions of Fiji (Fig 1), with less than half residing in urban settings (Table 1). There was a slightly higher percentage of females than males. Participants’ ages ranged from 2 to 88 years with a median of 37 years. There was no significant difference in age between males and females (*P* = 0.986) and the average number of eligible residents per household was 6.3.

**Table 1:** Demographic characteristics of respondents, and LF MDA coverage, in the Central, Northern, and Eastern Divisions of Fiji.

Variable	Category	Frequency	Percentage*	95% CI*	Coverage*	95% CI*	*P*-value
Division	Central	176	63.1	58.6–67.4	89.2	83.2–93.2	0.157
	Northern	64	8.4	4.5–15.0	95.7	85.0–98.9	
	Eastern	64	28.5	25.5–31.7	97.2	84.2–99.6	
Residence	Urban	128	45.6	41.8–49.5	87.2	79.1–92.5	**0.026**
	Rural	176	54.4	50.5–58.2	95.5	90.4–97.9	
Sex	Female	169	53.2	45.0–61.2	91.6	85.3–96.3	0.80
	Male	134	46.8	38.8–55.0	92.5	85.2–95.4	
Age	<10 year	21	8.5	5.1–13.6	78.9	53.8–92.3	0.308
	10–19 year	42	17.5	11.6–25.7	96.4	83.7–99.3	
	>19	241	74.0	65.9–80.7	92.1	87.0–95.3	
Household size	<5 persons	125	21.5	16.8–27.1	89.2	77.5–95.1	0.179
	5–8 persons	155	69.2	61.6–75.9	90.1	84.8–94.6	
	9≥ persons	23	9.3	4.7–17.7	96.8	79.9–99.6	

*Weighted based on the proportion of sub-divisional per divisional population size.

### LQAS for the 19 lots

Of the 19 lots tested by LQAS, seven were rejected, because at least two persons did not swallow their drugs, and were classified as having unacceptable coverage. In four (the Lami and Makoi medical areas of the Suva sub-division, and the Naitasiri and Macuata sub-divisions) of the seven rejected lots, the reported administrative coverage levels were above 80%; whereas in four (the Lakeba, Lomaiviti, Cakaudrove, and Tailevu sub-divisions) of the 12 accepted lots, administrative coverage levels were below 80% (Table 2). In two of the rejected lots (Makoi medical area and Macuata sub-divisions) the reported administrative coverage was above 100%.

**Table 2:** Results of the LQAS on sub-divisional LF-MDA coverage in the Central, Northern, and Eastern Divisions of Fiji.

Division	Sub-division	Medical area	Population > 2 years old	Administrative Coverage	Have you swallowed LF-MDA drugs?			
					Yes	No	DK/NR	D
Central	Suva	Suva	14,324	85.5%	15	1	0	A
Central	Suva	Raiwaqa	29,697	79.0%	13	2	1	R
Central	Suva	Samabula	16,297	90.6%	16	0	0	A
Central	Suva	Nuffield	44,618	58.6%	14	2	0	R
Central	Suva	Lami	28,282	83.9%	14	2	0	R
Central	Suva	Makoi	27,141	107.0%	12	3	1	R
Central	Suva	Valelevu	51,889	60.6%	13	3	0	R
Central	Rewa	-	77,504	84.8%	16	0	0	A
Central	Naitasiri	-	18,872	83.8%	12	4	0	R
Central	Tailevu	-	19,776	85.4%	16	0	0	A
Central	Serua-Namosi	-	30,369	91.0%	15	1	0	A
Northern	Bua	-	15,284	92.1%	16	0	0	A
Northern	Cakaudrove	-	30,077	62.8%	16	0	0	A
Northern	Taveuni	-	16,401	89.8%	15	1	0	A
Northern	Macuata	-	65,681	101.3%	14	2	0	R
Eastern	Kadavu	-	11,805	77.4%	16	0	0	A
Eastern	Lomaiviti	-	15,142	65.3%	15	1	0	A
Eastern	Lomaloma	-	2,514	109.3%	15	1	0	A
Eastern	Lakeba	-	3,494	73.8%	16	0	0	A
All			519,167	82%	279	23	2	

DK/NR: Don't know/No response; D: Decision; A: Accepted; R: Rejected.

### Surveyed program coverage and reasons for not participating in LF-MDA

The estimated LF-MDA program coverage for the 3 divisions was 92% (95% CI: 87.4–94.7). This is higher than the reported administrative coverage of 82% [27]. There was no significant difference in coverage estimates across divisions, sexes, age groups, or household size categories (Table 1). The estimated program coverage for the urban population was significantly lower than that for rural residents (*P* = 0.026).

**Fig 3:**
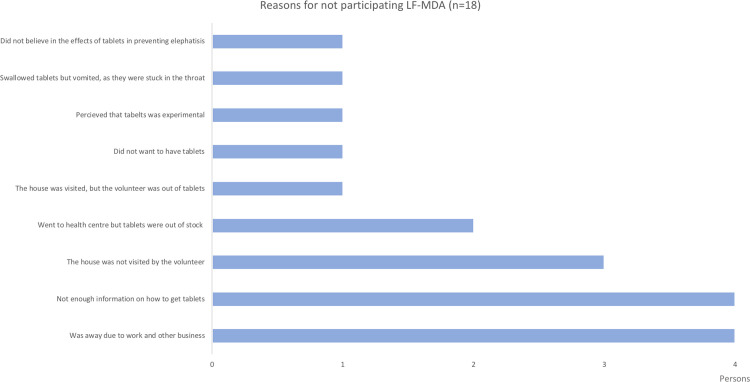
Number of people with reasons for non-participating in the LF-MDA in the Central, Northern, and Eastern Divisions of Fiji.

The most common reasons for persons not participating in the LF-MDA were that they were absent during the house-to-house drug distribution, that they did not have enough information about collection days/sites, or that the drug distributors did not visit their homes/community. (Fig 3).

### Participants’ experience with the LF-MDA drug distributors

At least nine out of 10 of the 304 surveyed participants claimed to have swallowed the medicine during the house-to-house distribution process (Table 3). About 88% could recall receiving the volunteers in their homes, and 98% were offered the drugs. More than ten percent of those participants who were offered the drugs claimed to have not received sufficient information on the medications from the drug distributors. Most of the participants (96%) verbally confirmed having swallowed the drugs, with 65% further recalling doing it in the presence of the drug distributors, and a further 59% remembering their fingernail being marked with indelible marker. About one-third felt unwell or weak after swallowing the drugs. Overall the delivery strategy for the 10^th^ round of LF-MDA was considered satisfactory by community members, but the level of satisfaction was significantly lower in the group that missed out on the LF-MDA (*P* = 0.037). Nine of the 22 unsatisfied participants interviewed would prefer to collect their medications from health centers, six requested to receive it from their homes, and two would prefer medications dispensed at the workplace or school instead of the home. One participant agreed with the strategy but felt that more detailed explanations needed to be provided by the drug distributors.

**Table 3:** Participants’ experience with the 10^th^ round of LF-MDA in the Central, Northern, and Eastern Divisions of Fiji.

Characteristic	Percent*	95% CI*
Swallowed the drugs (n = 279)	91.7	87.4–94.7
• Via house-to-house visit (n = 253)	90.5	85.5–93.9
• Distributed via other places (workplaces, markets, or health centers) (n = 26)	9.5	6.1–14.5
My house was visited by the volunteer (n = 266)	88.1	83.2–91.7
• Drugs were delivered and offered by the volunteer (n = 259)	97.9	95.2–99.1
• Drugs were not delivered (n = 7)	2.1	0.9–4.8
• The volunteers provided enough explanations at their visit (n = 236)	87.8	78.7–93.4
• No, they did not provide enough explanations (n = 28)	12.2	6.6–21.3
I swallowed the drugs when offered by the volunteer who visited the house (n = 249)	96.2	92.3–98.1
• My swallowing was directly observed (n = 165)	64.5	55.1–72.9
• My swallowing was not directly observed (n = 82)	34.8	26.4–44.2
• Don't recall (n = 2)	0.7	0.2–2.9
• They marked my nail (n = 154)	59.1	49.4–68.1
• They did not mark my nail (n = 93)	40.3	31.3–50.0
• Don't recall (n = 2)	0.6	0.1–3.3
Felt unwell after swallowing the drugs (n = 105)	35.9	28.5–43.9
Satisfied with the LF-MDA strategy (n = 279)	92.3	87.9–95.2

*Weighted based on the proportion of sub-divisional per divisional population size.

**Fig 4:**
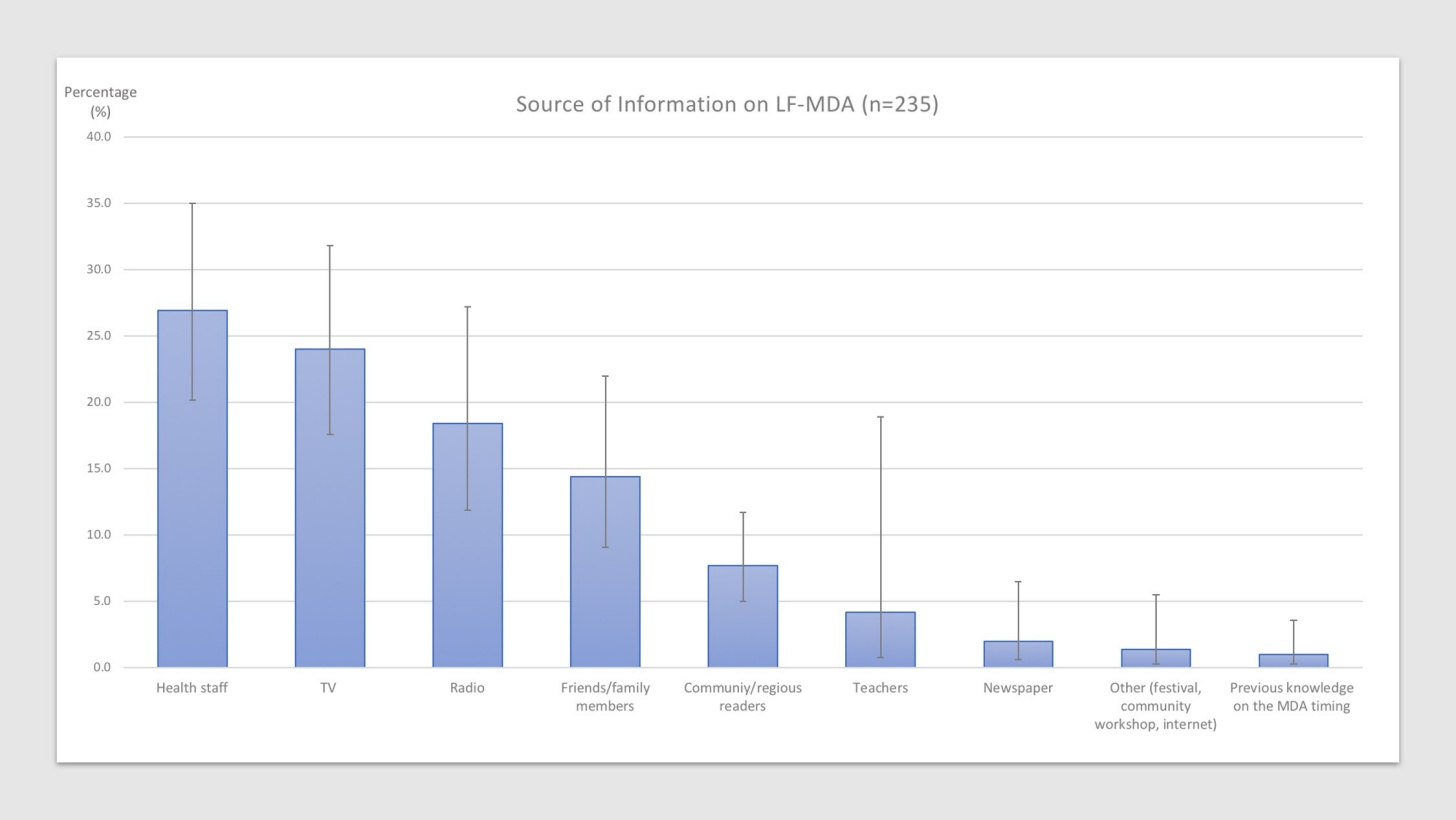
Sources of information on LF-MDA in the Central, Northern, and Eastern Divisions of Fiji by percentage*. *Proportion and confidence intervals were weighted based on the proportion of sub-divisional per divisional population size.

Almost all (90%) of the interviewees knew the purpose of the drug distribution, and 70% were aware of the complications associated with lymphatic filariasis (Table 4). A further 77% knew about the transmission of the disease by mosquitoes. However, there was no significant difference in the level of knowledge between participants that swallowed the drugs and those who did not. At least 86% of the participants could recall receiving information about the LF-MDA round before the distribution (Table 4). The sources of information about the LF-MDA round are presented in Fig 4.

**Table 4:** Survey respondents’ levels of knowledge, practice, and attitude associated with LF-MDA in the Central, Northern, and Eastern Divisions of Fiji.

Characteristic	Percent*	95% CI*	*P-*value
Knew what the LF-MDA drugs were for (n = 274)	90.0	82.0–94.7	
Among those who swallowed (253/279)	90.9	81.9–95.6	0.5627
Among those who did not swallow (21/25)	81.0	56.9–93.2	
Knew what the complications of LF were (n = 202)	69.6	61.8–76.4	
Among those who swallowed (186/279)	70.1	61.7–77.3	0.0568
Among those who did not swallow (16/25)	63.8	41.3–81.5	
Knew how LF is transmitted (n = 231)	77.0	68.7–83.6	
Among those who swallowed (211/274)	75.9	67.1–83.1	0.091
Among those who did not swallow (20/24)	89.1	73.1–96.1	
Informed about the LF-MDA round beforehand (n = 243)	85.7	80.0–90.0	
Among those who swallowed (226/274)	87.1	81.2–91.3	0.1503
Among those who did not swallow (17/25)	71.2	48.0–87.0	

*Weighted based on the proportion of sub-divisional per divisional population size.

## Discussion

Population-based assessment of post-MDA coverage is an essential tool for measuring the effectiveness of MDA interventions and exploring the barriers to services [28]. These surveys provide practical information for decision makers that is not dependent on population data, which may not always be reliable [29]. We conducted a survey to determine which medical areas in Suva and the sub-divisions in the rest of the 3 divisions targeted by the LF-MDA 10^th^ round did not achieve at least 80% program coverage. Despite an overall high LF-MDA program coverage, the LQAS results revealed the existence of pockets of low coverage at medical and sub-divisional levels: more than a third of the 19 lots were identified as areas with probable low coverage; this information could provide the basis for strategies to increase coverage and reach targets.

Most of the lots identified as having unacceptable coverage, except the Naitasiri sub-division, were in urban areas: five medical areas in Suva, the capital city, and the other sub-division, Macuata, which is an urban centre of the 2nd largest island. This is in line with the findings from other studies showing greater difficulty in achieving LF-MDA coverage goals in urban canters than in rural ones [30–32, 33]. The presence of non-resident populations, limited ability of the urban dwellers to receive door-to-door treatment, high awareness of the infection but low perception of its risks, and low compliance due to insufficient information, have been identified as barriers to MDA success in urban areas [30]. Hence, it is useful to look at the disaggregated data for urban settings, as we did in our study, since the evidence shows that a large number of unreached targets often could reside especially in rapidly expanding urban slums [34]. In contrast to urban areas, in the Naitasiri sub-division, which is another failed lot, and the surrounding sub-division of the capital Suva, it is thought that a large portion of the community members were covered in Suva [23, 35], where they spend most of their time. Hence, we believe that the actual level of service uptake by the residents in that area was low. However, we cannot rule out the possibility that other variables such as inaccurate registration of dose delivered (numerator) could act as the main source of the discrepancy, given that we did not assess the quality of the data on site [21]. In this regard, it would be helpful to explore additional indicators, such as the number of households registered, and the number of persons who swallowed the medication as a function of each drug distributor, to see whether the programme has run efficiently in these low performing areas rather than relying solely on coverage levels [21].

Discordance between the reported administrative coverage and survey results has been documented not only in MDA rounds but also in mass vaccination campaigns [28, 36, 37]. In our study, levels of administrative coverage in these failed lots ranged from 59% to 107%, and more than a half of them had a reported coverage higher than the national target of 80%, implying that relying on the reported coverage for mop-up decisions may not reflect the real situation. Imprecise population estimates (denominator) at lot level due to movement of populations across lot borders, which can be aggravated in fast growing urban cities, and outdated censuses can affect the accuracy of the reported coverage figures [38, 39, 40]. Adapting numerator-based data to avoid imprecise denominators for marginalised populations [34], as well as caution in using solely administrative coverage levels for assessing programme performance has been widely recommended [29] as complementary measures.

Cluster surveys are used to estimate programme coverage with point estimates, while LQAS has been used to classify coverage (i.e., generate labels like acceptable/not acceptable) [39]: LQAS can provide critical information on pockets of areas not necessarily fully covered by the intervention with a smaller sample size [12]. Depending on levels of expected coverage, cluster sampling survey methods require sample sizes of the order of hundreds of individuals to obtain reliable estimates even for the districts surveyed [19, 21]. For instance, if we had identified pockets with low coverage using standard 30*7 cluster samplings, as we did in our study, either at the medical area or sub-divisional level, we would have needed at least 19*210 participants, which is 10-fold our actual sample size. Instead, nesting LQAS in the survey or aggregating data from LQAS enables one to conduct the study with greater flexibility and obtain more detailed information by area than with 30-cluster surveys [38]. In this study, LQAS was embedded in a two-stage random sample to assess LF-MDA program coverage at the sub-population level. Selecting a little more than three hundred participants as the sample actually increased the feasibility of the survey in the field and provided area-specific information that could identify pockets with lower coverage.

The present study adapted the standard LQAS technique that has been applied to several public health programmes, as it was planned before the recent global recommendations for rapid monitoring of MDA coverage, and coverage evaluation surveys, were made [19–21]. In fact, our survey design is closer to the supervisor’s rapid coverage monitoring tool [20] than the 30-cluster coverage survey [19], and the only difference is the sample size of 16 rather than 20 [20]. This rapid monitoring tool was designed to maximize the benefits of LQAS: identifying areas of weak coverage with statistical reliability, differentiating areas of varying coverage with greater precision, and allowing for trend analysis of intervention quality [13, 41, 42]. In the study, we proved that this is a practical methodology since the interpretation of the results is straightforward and immediate and can be further facilitated by using mobile phones in the field to improve the timeliness of data collection and reporting [16, 41]. LQAS is useful in addressing remaining gaps in programme quality, since it helps focus resources on high-risk areas to prevent the continued transmission of infectious agents [13, 43]. To our knowledge, this is one of only a small number of studies applying LQAS to assess the coverage of a nation-wide round of LF-MDA [37].

We also evaluated several aspects of the LF-MDA implementation strategy by exploring levels of drug compliance, reasons for not taking the medication, and the extent of penetration of social mobilization messages. If verbal reports by participants are considered a reliable source of information about the receipt of drug treatment [44], then the post-LF-MDA drug coverage estimated by this survey was 92%, which is higher than the reported administrative coverage of 82% [27]. This implies that the 10^th^ LF-MDA round in Fiji achieved the desired coverage goal of 80% set by the national programme. Based on the responses of the survey respondents, the most common reasons for non-participating in the LF-MDA were being absent during the time of drug distribution and being unaware of the LF-MDA round itself. Other reasons for not swallowing the medication were either that the drug distributors did not reach the household, or they had run out of drugs by the time they visited the household due to stock-out. This is similar to the finding from other studies that the main determinant of low drug uptake in low-resource settings was not refusals, but rather problems with service organization, such as awareness of MDA and the length of the drug distribution period [36, 38]. Alternative options should be planned for the absentees, such as designating nursing stations or health centers as fixed sites to provide drugs for those who missed the visits, and these should be available before, during and after the LF-MDA period.

A little more than 90% of participants responded positively concerning the level of satisfaction for the LF-MDA strategy. Disappointingly, more than 1 out of 10 drug distributors did not provide the survey respondents with sufficient explanation. Moreover, only 65% of the volunteers observed the drug swallowing and only 60% marked the fingernail thereafter. The reasons for not observing drug swallowing were possibly: (1) cultural difficulty faced by the drug distributors when refusing requests to leave medications for family members who were absent at the time. Despite training in mock exercises, it was a challenge for mainly young adolescent drug distributors to politely refuse an older adult requesting medication for family members in absentia; and (2) the past LF-MDA practice of leaving medication for family members in absentia was difficult to change, especially when previous drug distributors were not required to directly observe swallowing. In an attempt to mitigate this situation, mass media messages were communicated during the pre-LF-MDA sensitization period about leaving ‘no tablets behind’. However, since less than half the survey participants learned about the LF-MDA round from these media and a greater proportion learned of it through word of mouth, the low directly observed swallowing rate is an indication that the message may have not permeated well. Another attempt was made by marking fingernails, as commonly used in polio vaccination campaigns. The uptake of this strategy was limited to two-thirds of the participants, highlighting the unfamiliarity with the procedure and the need to improve the quality of training for drug distributors in practicing it.

The 2% of participants who claimed to have not been given the medications during the LF-MDA period were supposed to be re-visited by drug distributors who ran out of medications. Despite the instruction to revisit the houses after replenishing the medication supply, the distributors possibly chose instead to move quickly to other localities. These events were not uncommon especially with the shortage of albendazole in the field: initially, all volunteers were given a bottle of 50 mg DEC containing 1,000 tablets and another bottle with a 100 400 mg albendazole tablets and were instructed to return to a health center for replenishment. The quantity of DEC would have been sufficient to cover at least 120 people, but they would run out of albendazole after 100 residents, sooner than after DEC. Since most of the distribution by drug distributors was through house-to-house visits rather than by fixed-site distribution, the quantities packaged are likely to affect the individual distributor’s performance. Hence, in future LF-MDA rounds it would be important to have a sufficient level of buffer stocks at the periphery, or to re-package medications in matched amounts rather than 1 bottle each, when the strategy requires a high level of involvement of individual drug distributors providing house-to-house distribution.

The overall levels of knowledge on LF and its complications of the survey respondents were high, and no difference was observed between those that swallowed the drugs and those that did not. More than 80% of the participants were aware of the LF-MDA round in advance and the figure was slightly higher for those who swallowed the drugs, which showed good permeation of information to the public. The most common source of information on the LF-MDA round were health workers, indicating the importance of the active engagement of the health workforce in the successful implementation. Although the main messages communicated during the LF-MDA were not explored in detail in our study, it could be more effective to further stress drug distribution strategy-related information such as dates of distribution, and locations of pick-up stations when visits are missed. Moreover, it is becoming increasingly popular to adopt social network services (SNS) to further advertise public health initiatives and programs. SNS have the potential to extend the reach and efficiency of essential public health services by offering a range of multidirectional communication strategies and interactions to plan and deliver LF-MDA-related messages effectively to the public [45]. SNS should be considered in future LF-MDA rounds.

This study has several limitations. First, recall bias by the survey participants will not be negligible, given that it was conducted five months after the LF-MDA round. A possible explanation for the higher value of the estimated program coverage than the reported administrative coverage is linked to the fact that we relied on verbal reports, which were not validated via review of the registration booklets. People tend to say yes when they know there is a possibility of stigmatization if they say no. Hence, in order to reduce such effects, it is recommended to undertake the evaluation within one month of the end of the LF-MDA round and to introduce tools for possible verification of verbal reports, such as review of booklets, fingernail marking or individual drug treatment cards. The fingernail mark used in this study was detected in 60% of the treated population, and other marks may have faded by the time the survey was conducted. Other areas of improvement include the introduction of an "MDA card" similar to an immunization card, which is not routinely introduced for MDA mainly due to the associated costs. Second, there may be a correlation between being at home during the visit of the LF-MDA drug distributors and being at home for the interview, which could lead to over-estimation of the coverage. Using different sampling methods that are not household-based could reduce this bias. In addition, this assessment is based on surveying ‘customers’ satisfaction’ with the delivery of services. Additional valuable and meaningful information could be gained by conducting focus group interviews or narrative surveys with the drug distributors who were involved in drug distribution [46] or the health staff who supervised them, to investigate the providers’ point of view [47]. Lastly, the study was designed before the global recommendation for rapid monitoring of MDA coverage was made available and, as for LQAS, we applied a smaller sample size with relatively large alpha and beta levels than other studies [16, 21]. This may have led to incorrect conclusions as to whether coverage levels of individual lots were acceptable or not. However, we formulated the null hypothesis towards the conservative side, by assuming less program coverage for the intervention, and requiring strong evidence before rejecting the null hypothesis [16]. In this way the decisions made are in the direction of protecting the population concerned.

## Conclusion

Our data indicate that pockets of low coverage existed, based on the LQAS methodology, which provided action-oriented data at the local level (medical areas or sub-divisions). Drug compliance and satisfaction of the survey respondents was high, even after repeated LF-MDA rounds. Hence, a key recommendation is to correlate findings with periodic future LF-MDA rounds and to strengthen service delivery and logistic arrangements in the lots with low coverage. Conducting a timely coverage assessment, such as a focal LQAS survey in these areas with a previous history of low performance is warranted, in order to be able to organize timely corrective action.

## Supporting information

S1 Data (XLSX)
